# DOM degradation by light and microbes along the Yukon River-coastal ocean continuum

**DOI:** 10.1038/s41598-021-89327-9

**Published:** 2021-05-13

**Authors:** Brice K. Grunert, Maria Tzortziou, Patrick Neale, Alana Menendez, Peter Hernes

**Affiliations:** 1 Department of Earth and Atmospheric Sciences, The City College of New York, The City University of New York, 160 Convent Avenue, New York, NY 10031 USA; 2 Smithsonian Environmental Research Center, 647 Contees Wharf Rd, Edgewater, MD 21037 USA; 3 Department of Land, Air and Water Resources, University of California, Davis, CA 95616 USA

**Keywords:** Biogeochemistry, Carbon cycle, Ecology, Carbon cycle

## Abstract

The Arctic is experiencing rapid warming, resulting in fundamental shifts in hydrologic connectivity and carbon cycling. Dissolved organic matter (DOM) is a significant component of the Arctic and global carbon cycle, and significant perturbations to DOM cycling are expected with Arctic warming. The impact of photochemical and microbial degradation, and their interactive effects, on DOM composition and remineralization have been documented in Arctic soils and rivers. However, the role of microbes, sunlight and their interactions on Arctic DOM alteration and remineralization in the coastal ocean has not been considered, particularly during the spring freshet when DOM loads are high, photoexposure can be quite limited and residence time within river networks is low. Here, we collected DOM samples along a salinity gradient in the Yukon River delta, plume and coastal ocean during peak river discharge immediately after spring freshet and explored the role of UV exposure, microbial transformations and interactive effects on DOM quantity and composition. Our results show: (1) photochemical alteration of DOM significantly shifts processing pathways of terrestrial DOM, including increasing relative humification of DOM by microbes by > 10%; (2) microbes produce humic-like material that is not optically distinguishable from terrestrial humics; and (3) size-fractionation of the microbial community indicates a size-dependent role for DOM remineralization and humification of DOM observed through modeled PARAFAC components of fluorescent DOM, either through direct or community effects. Field observations indicate apparent conservative mixing along the salinity gradient; however, changing photochemical and microbial alteration of DOM with increasing salinity indicate changing DOM composition likely due to microbial activity. Finally, our findings show potential for rapid transformation of DOM in the coastal ocean from photochemical and microbial alteration, with microbes responsible for the majority of dissolved organic matter remineralization.

## Introduction

The major Arctic Rivers—Ob’, Yenisey, Lena, Kolyma, Yukon and Mackenzie—deliver over 18 Tg of terrestrially-derived dissolved organic carbon (DOC) per year to the coastal ocean, with nearly half of this material delivered during the spring freshet^1^. Long-term observations in Arctic rivers have confirmed increases and a recent acceleration in total discharge accompanying rapid warming in the Arctic^2,3^ with concurrent increases in total DOC export^1^. This intensification of the hydrologic cycle and altered flow paths from thawing permafrost affects both the timing and composition of organic matter delivered to aquatic systems^4,5^; however, how these factors will impact dissolved organic matter (DOM) cycling in Arctic rivers and the coastal ocean is not known. DOM is an important source of carbon and nutrients for microbes in Arctic ecosystems^6,7^, with use and remineralization of DOM dependent on its composition^8,9^. Interactive exposure to sunlight and processing by the microbial community can alter DOM composition and energy content, with continued re-working of the DOM pool contingent on microbial community composition and enzymatic capabilities^10,11^. Studies in Arctic soils and rivers have shown competitive degradation of high molecular weight, aromatic molecules between sunlight and microbes^12,13^, significant contributions of microbial metabolic byproducts to total DOC^14^, and increasing DOM bioavailability during transport through river networks^15^. However, what drives transformation and remineralization of DOM from Arctic rivers in the coastal ocean is not well understood, particularly during the spring freshet when DOM loads are high and residence time across aquatic systems is low.

DOM, and particularly the chromophoric fraction, CDOM, is the dominant bio-optical constituent of Arctic and sub-arctic surface waters, impacting light and nutrient availability, sea ice cover and ecosystem productivity^16–19^. In Arctic Rivers, terrestrial material, including lignin and hydroxy-benzene biomarkers, increases as a DOM source during the spring freshet^7,20^. Lignin concentrations correlate strongly with total DOC and CDOM absorption (a_CDOM_(λ), λ denotes light wavelength) across the major Arctic Rivers^21,22^, and tannic compounds constitute a significant carbon pool available for photo- and microbial alteration^12,13^. Lignin and partially oxidized lignin derivatives are traditionally seen as relatively refractory DOM, with North Atlantic Deep Water sourced from the Arctic Ocean still exhibiting elevated concentrations of lignin and optical properties consistent with terrestrial CDOM^23,24^. Processing of high molecular weight terrestrial material, including lignin, is tied to proximity to source, specific enzymes, and microbial cell size^25,26^. The dependence of DOM processing and composition on larger cell-sized microbes has been documented, with high nucleic acid and larger cell sized microbes from an Arctic fjord responsible for the majority of terrestrial DOM processing^26^. The size-fractionation of microbes has also been shown to impact DOM composition, with a larger size fraction encompassing the entire microbial community in mesocosm experiments resulting in DOM composition more representative of that observed in the environment^27^. These effects point to the role of specific microbial functional traits, clades, and community effects, that ultimately shape the transformation of DOM and highlight the role of larger microbes in DOM processing^9,28^.

Microbial community composition and group abundance have explained the majority of variability in DOM assimilation in Arctic waters, indicating the importance of the entire microbial community in DOM degradation. Spatial variability in DOM processing in the coastal ocean is expected, as microbial community composition can vary strongly along shelf to open ocean transects in the Arctic^29,30^. Prior work has shown that environmental conditions, including temperature and salinity, strongly regulate microbial structure and function^31,32^. DOM source, as well as the metabolic capability of distinct microbial groups and interactive effects within the microbial community and with sunlight also play a strong role in terrestrial DOM use and remineralization across aquatic environments^11,33^.

Building on this past work, we hypothesized that: (1) photochemical exposure would increase net remineralization of DOC and expand available pathways for degradation of DOM by microbes; and (2) larger cell size bacteria (those uniquely present in the 0.7–3.0 μm fraction here) would more readily degrade high molecular weight (HMW) terrestrial DOM. We tested these hypotheses by observing CDOM and DOC variability in situ and assessing the impact of photochemical transformation, microbial processing and their interplay on CDOM and DOC amount and quality along the Yukon River freshwater-marine continuum—one of the six major rivers draining the terrestrial Arctic—during peak spring discharge (Fig. S1). We manipulated DOM using three experiments explicitly characterizing photochemical, microbial, and microbial after photochemical transformation of DOM, with DOM compositional shifts assessed via changes in DOM optical properties (CDOM and fluorescent DOM) and DOM concentration assessed through changes in DOC. Dark microbial incubation experiments were treated with < 0.7 and < 3.0 μm size-fractionated inocula to explore the role of different microbial size classes on DOM transformations, following the hypothesis that the < 3.0 μm inoculum would more readily degrade HMW terrestrial DOM due to the inclusion of larger microbes as well as microbes present in all other inocula^26^.

## Results and discussion

### Photochemistry alters microbial bioavailability of DOM

**Figure 1:**
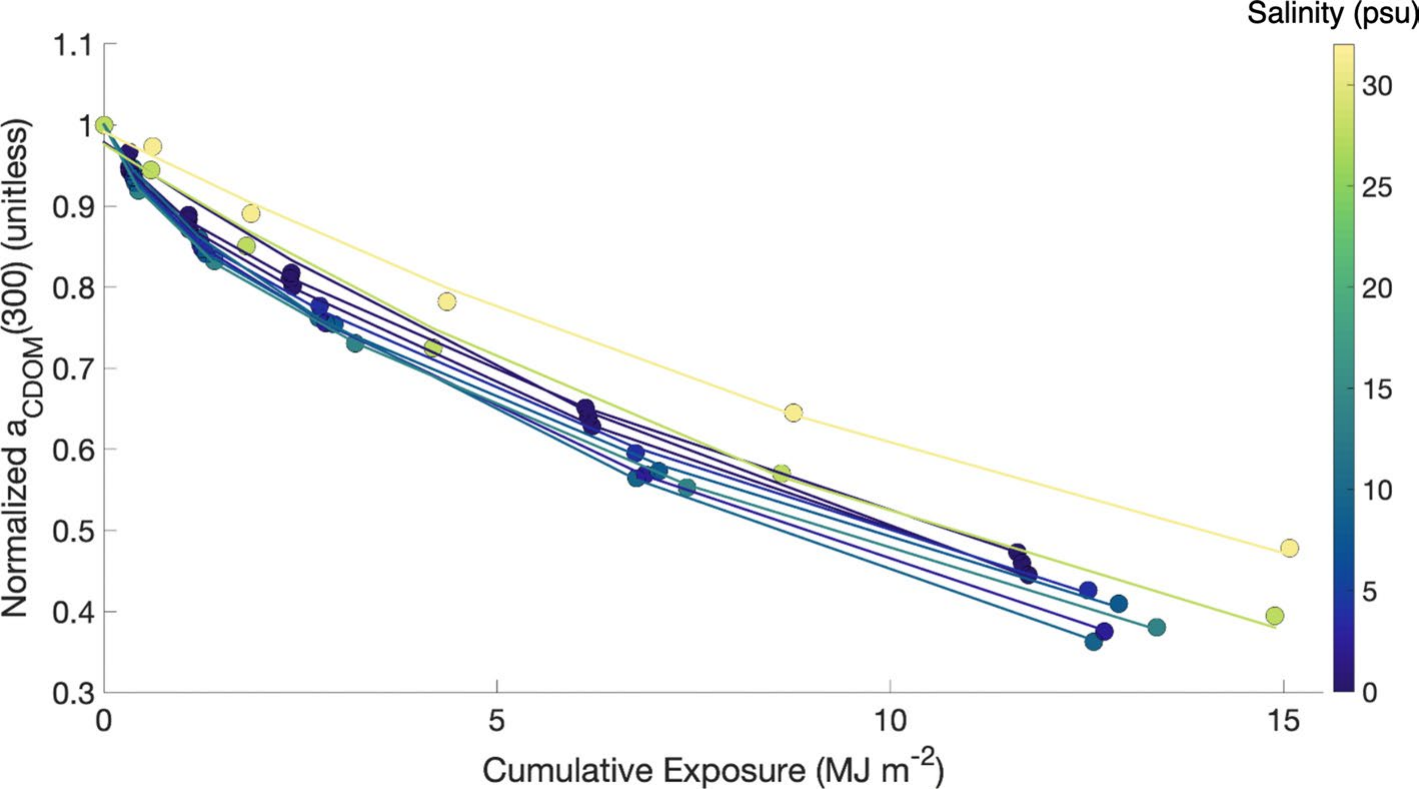
Mean observed (colored circles) and modeled (solid line) photochemical loss of a_CDOM_(300) for each station, normalized to the initial a_CDOM_(300) value for scaling purposes. Marker and line color indicates the mean salinity observed in the upper 1 m of the water column at each station. Photochemical loss of CDOM was modeled with a single exponential for stations 10, 12 and 24, and a double-exponential for all other stations (Table S1). Peak absolute loss of absorption occurred between 290 and 305 nm for all stations and time points.

Photochemical processing significantly altered the molecular structure of DOM and increased available microbial processing pathways, with a far greater impact on DOM composition (> 40% loss of CDOM across the Yukon salinity gradient) than on DOM concentration (represented by 16.9–21.7% DOC loss). CDOM also displayed two distinct photoreactivities across most stations, as river and plume stations (lower salinities) were consistently well-modeled with a double exponential decay function (excluding St 10, the main river mouth) and oceanic (higher salinity) stations were well-modeled with a single exponential decay function (Fig. 1; Table S1). Cumulative UV exposure during the course of the experiments ranged from 11.61 MJ m^−2^ for St 6 (salinity = 0.1 psu) to 15.08 MJ m^−2^ for our oceanic end-member St 12 (salinity = 31.2 psu). Not accounting for self-shading, this exposure ranged from 61 to 69% of incident solar irradiance, consistent with average UV exposure in the first optical depth for clear water conditions (see “Supplementary Methods”). After 24 days of UV exposure, average DOC change in the 0.2 μm filtrate was − 21.7 ± 6.6% for river stations and − 16.9 ± 1.5% for plume stations (Table 1). Our photochemical incubations included the influence of ultra-microbacteria that passed through a 0.2 μm filter (see “Methods”). After accounting for observed changes to a_CDOM_(350) and DOC in the < 0.2 μm dark treatment (losses of 1–9.8% and 11.3–17.0%, respectively; Table 1), photochemical processing influenced DOM composition (39.1–65.7% loss in CDOM across sites) far more than concentration (DOC removal of 4.7–5.6%). Although microbial activity can be inhibited by UV exposure and formation of reactive oxygen species^13,34^, our experiments used a low UV irradiance (5.6 W m^−2^) to limit such effects (see “Methods”). The net remineralization of DOC was not significantly different between the < 3.0 μm dark treatment and the 3.0 μm-after-PB experiment, further indicating that some of the DOC loss in the PB experiments was due to microbial activity. DOC loss over our 24 day incubations was consistent with short-term photoincubation experiments in the coastal Arctic^35^, suggesting that much of the DOC removal occurred early in our experiments.

**Table 1:** Change in dissolved organic carbon concentration (%), fluorescent components (%), CDOM absorption at 350 nm (a_CDOM_(350); %) and spectral slope from 275–295 and 350–400 nm (absolute change, nm^−1^) from day 0 to day 24.

Parameter	Loc	PB	0.2 μm	0.7 μm	3.0 μm	3.0 μm-after-PB
DOC	R	− 21.7 ± 6.6%	− 17.0 ± 12.6%	− 15.2 ± 2.2%	− 20.0 ± 8.7%	− 21.0 ± 11.4%
	P	− 16.9 ± 1.5%	− 11.3 ± 10.0%	− 12.4 ± 5.4%	− 16.6 ± 7.6%	− 17.0 ± 7.2%
	O	− 27.2 ± 1.1%		− 11.2 ± 0.1%	− 10.0 ± 0.1%	− 7.7%
C1	R	− 83.3 ± 0.6%	− 1.4 ± 1.8%	− 0.1 ± 1.4%	1.7 ± 1.6%	4.6 ± 4.2%
	P	− 85.5 ± 0.7%	1.4 ± 2.5%	0.4 ± 2.8%	2.9 ± 2.4%	14.5 ± 3.7%
C2	R	− 64.7 ± 1.3%	− 2.5 ± 2.1%	0.0 ± 0.7%	1.3 ± 1.5%	5.2 ± 2.6%
	P	− 65.1 ± 1.9%	1.3 ± 3.4%	2.0 ± 4.2%	3.5 ± 4.2%	6.7 ± 3.0%
C3	R	− 80.0 ± 0.8%	− 0.9 ± 1.7%	0.7 ± 1.1%	2.5 ± 1.5%	8.7 ± 2.6%
	P	− 82.9 ± 1.1%	2.1 ± 2.9%	0.7 ± 3.2%	3.5 ± 2.7%	13.8 ± 3.5%
C4	R	− 46.0 ± 0.8%	− 1.9 ± 3.8%	0.7 ± 2.2%	10.7 ± 2.1%	− 9.2 ± 1.0%
	P	− 48.9 ± 1.2%	− 0.6 ± 3.6%	− 0.3 ± 4.1%	6.0 ± 2.6%	− 3.8 ± 4.0%
C5	R	− 9.0 ± 3.6%	0.4 ± 1.3%	0.7 ± 1.7%	2.8 ± 1.8%	− 16.4 ± 6.4%
	P	− 21.8 ± 4.7%	− 7.9 ± 10.4%	− 10.2 ± 9.9%	− 8.8 ± 10.6%	− 5.9 ± 9.6%
C6	R	171.0 ± 20.5%	9.5 ± 7.0%	10.9 ± 10.4%	21.8 ± 8.3%	− 31.0 ± 2.1%
	P	153.0 ± 39.2%	5.4 ± 19.7%	0.4 ± 12.1%	15.6 ± 16.4%	− 16.9 ± 12.7%
a_CDOM_(350)	R	− 58.4 ± 1.7%	− 5.7 ± 1.3%	− 1.5 ± 0.4%	− 2.3 ± 0.6%	− 7.4 ± 5.4%
	P	− 66.7 ± 3.2%	− 1.0 ± 8.0%	− 1.9 ± 1.9%	− 2.1 ± 0.9%	− 4.7 ± 4.1%
	O	− 48.9 ± 5.7%	− 9.8%	− 4.7%	− 10.5%	27.1%
**S**_**275:295**_						
T0	R	0.0140 ± 0.0000	0.0139 ± 0.0014			0.0168 ± 0.0002
T24		0.0239 ± 0.0007	0.0138 ± 0.0015	0.0138 ± 0.0002	0.0139 ± 0.0002	0.0170 ± 0.0003
T0	P	0.0151 ± 0.0020	0.0150 ± 0.0021			0.0182 ± 0.0017
T24		0.0272 ± 0.0022	0.0151 ± 0.0025	0.0149 ± 0.0020	0.0150 ± 0.0021	0.0188 ± 0.0024
T0	O	0.0251	0.0241			0.0257
T24		0.0358	0.0247	0.0242	0.0251	0.0270
**S**_**350:400**_						
T0	R	0.0175 ± 0.0001	0.0172 ± 0.0001			0.0154 ± 0.0001
T24		0.0157 ± 0.0002	0.0170 ± 0.0001	0.0170 ± 0.0002	0.0171 ± 0.0001	0.0153 ± 0.0001
T0	P	0.0175 ± 0.0004	0.0173 ± 0.0004			0.0163 ± 0.0007
T24		0.0158 ± 0.0006	0.0167 ± 0.0009	0.0172 ± 0.0005	0.0173 ± 0.0006	0.0155 ± 0.0004
T0	O	0.0177	0.0152			0.0171
T24		0.0161	0.0189	0.0178	0.0190	0.0152

Values include standard deviation, calculated between stations (river and plume) or between replicates (ocean). Where standard deviation is absent, the value was zero. Stations are delineated by location (Loc), delineated by salinity, as river (R; ~ 0 psu), plume (P; 2.7–27.8 psu) and ocean (O; 31.2 psu). The number of stations considered for each group was 3, 6 and 1, respectively.

**Figure 2:**
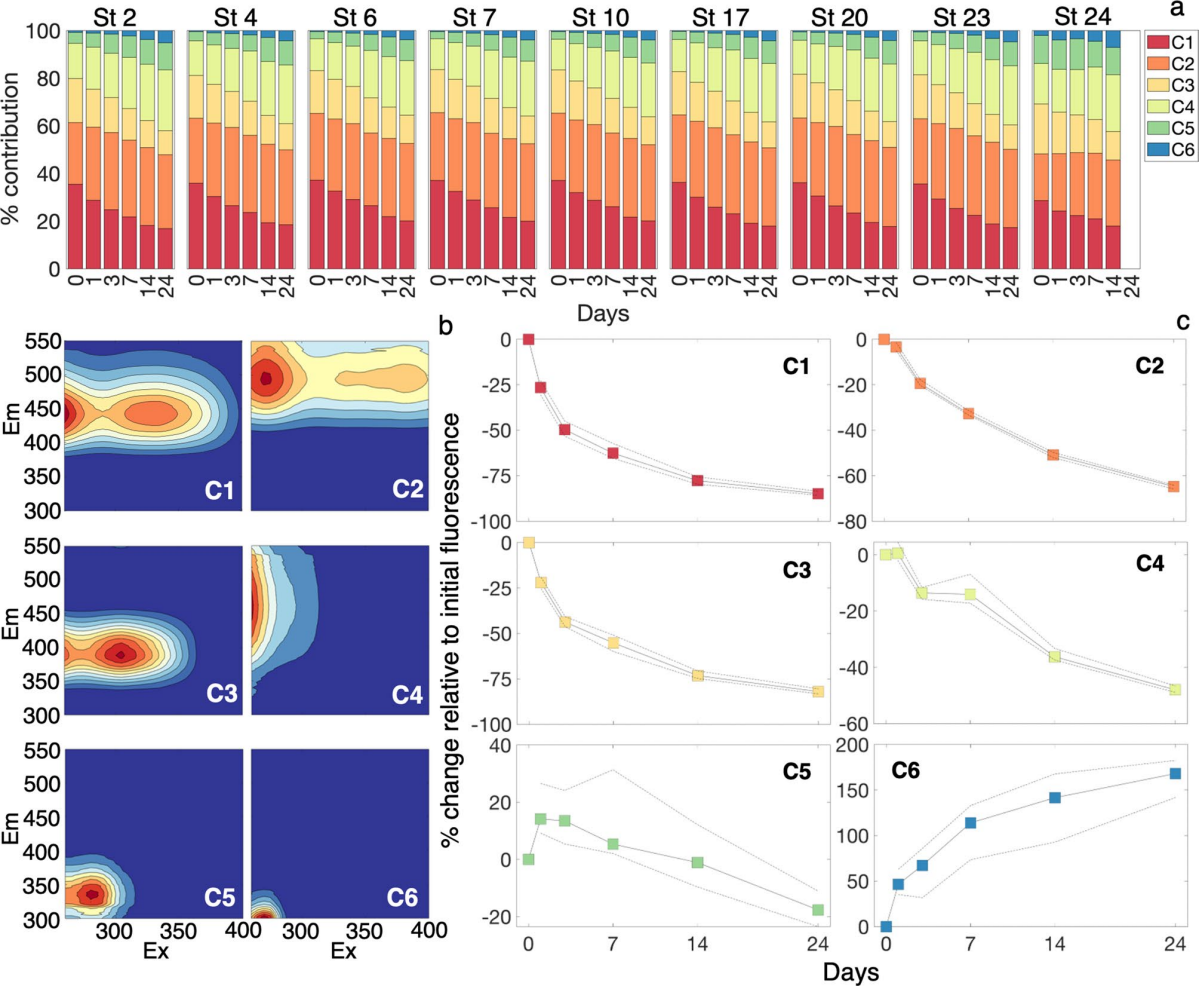
(**a**) PARAFAC component percent contribution to total fluorescence (Eq. 4) over the time course of photobleaching experiments. (**b**) Component fingerprints for the six component PARAFAC model. The same figure with more spectrally-resolved axes can be viewed in the “Supplemental Material” (Fig. S3). (**c**) Percent changes in individual PARAFAC components due to photobleaching. The solid lines and colored squares indicate the median change across all stations and replicates and the dashed lines indicate the first and third quantile ranges for all stations and replicates.

Our fluorescence results further supported our hypothesis that photochemical alteration increases microbial transformation pathways of DOM, primarily through photodegradation of humic DOM and formation of terrestrial derivatives (e.g., lignin depolymerization products). PARAFAC modeled fluorescent components displayed a consistent photochemical response across stations (Figs. 2; S2). We designated our six modeled components as visible humic (C1), long-wavelength humic (C2), UV humic (C3), degraded humic (C4), terrestrial derivatives (C5) and protein-like (C6). Increasing UV exposure led to decreasing contributions to total fluorescence of visible and UV humic components. Long-wavelength humics maintained relative contributions to total fluorescence in river stations (0 salinity) and increased in higher salinity samples, while degraded humic, terrestrial derivatives and protein-like contributions to total fluorescence increased (Fig. 2a). Visible, long-wavelength, UV and degraded humic fluorescence intensity decreased over time, relative to initial fluorescence intensity (Fig. 2c). Terrestrial derivative fluorescence intensity increased initially, then decreased, suggesting formation of this material followed by photodegradation. Protein-like DOM displayed increasing fluorescence intensity over time approaching a maximum, indicating this material is photochemically produced from a limited substrate pool (Fig. 2c). Photochemical production of this component has been observed in Arctic river DOM samples, DOM produced from photodegradation of terrestrial floc, as well as in open ocean samples, indicating a diversity of potential sources for this material^7,36,37^.

The uneven decrease of degraded humics (C4) suggests that this is the most heterogeneous component. C4 matched with 14 modeled PARAFAC components on OpenFluor and is consistent with Peak A described in the literature^38^. C4 was previously found to positively correlate with climate (mean annual temperature and length of growing season) and nutrients at high latitudes and is typically affiliated with degraded, terrestrially sourced DOM^22,39^. C4, C5 and C6 are all consistent with molecules derived from photochemical degradation of larger molecules, as evidenced by temporary (C4, C5) and consistent (C6) increases in fluorescence intensity over the course of the photobleaching experiment. C5 and C6, however, were more consistent with small aromatic molecules, with a defined fluorescent structure consistent with tryptophan and tyrosine fluorescence. C5 matched with 36 modeled components on OpenFluor and C6 matched with seven models, including a tyrosine standard^40^. Considering the high concentrations of lignin in the Yukon River^21,41^ and the disproportionate role of lignin, tannin and depolymerization products in CDOM optics, C5 and C6 are very likely reactive intermediaries from degradation of lignin and tannin^42–45^. Based on results in the microbial degradation experiments, formation of these compounds in photoexposure incubations can fundamentally alter microbial use of terrestrial DOM, effectively bypassing the necessity for creation of these materials in dark incubations.

### Microbial size dependency of DOM diagenesis

**Figure 3:**
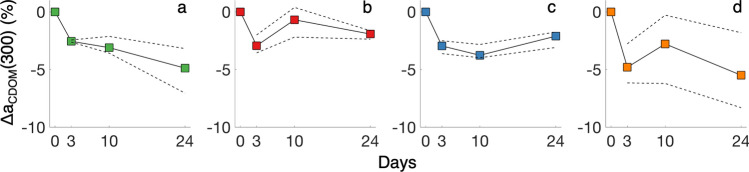
Median (solid line with colored squares) and interquartile range (dashed lines) for Δa_CDOM_(300) at each time point relative to initial a_CDOM_(300) for (**a**) 0.2 μm, (**b**) 0.7 μm, (**c**) 3.0 μm and (**d**) 3.0 μm-after-PB treatments.

**Figure 4:**
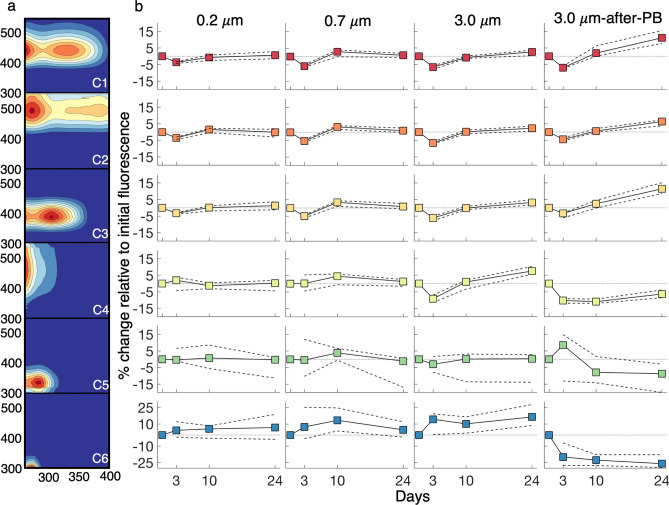
(**a**) Component fingerprints for the six component PARAFAC model, corresponding to component change in treatments in the right panel. (**b**) Median (solid line with colored squares) and interquartile range (dashed lines) for percent change in individual PARAFAC components (relative to initial component loading) for 0.2 μm, 0.7 μm, 3.0 μm and 3.0 μm-after-PB treatments. Percent change is relative to the initial fluorescence maximum for each component, indicating change in that component irrespective of change in other components.

Microbial transformations of DOM resulted in three predominant trends (Table 1): (1) The < 3.0 μm microbial size class removed more DOC in river and plume samples, indicating large microbes increase terrestrial DOC remineralization; (2) Relative bioavailability of DOM decreased with increasing salinity across all microbial size class inocula; and (3) Transformations of photoexposed DOM showed a distinct shift in how DOM is used by microbes after exposure to light. Both a_CDOM_(λ) and modeled PARAFAC components displayed a drawdown from day 0 to day 3, indicative of rapid removal of biologically labile humic DOM (Figs. 3, 4).

Changes to a_CDOM_(λ) were not consistent across or within treatments, characteristic of competing processes of consumption and production of CDOM by the microbial community (Fig. 3). The < 3.0 μm microbial size class displayed an eventual net humification of CDOM (increases in C1, C2 and C3), especially in the 3.0 μm-after-PB experiment (Fig. 4). All humic components showed similar kinetics during the incubations within treatments (Fig. 4b) despite visible and long-wavelength humics being attributed to terrestrial sources^38,46^ and UV humics traditionally being affiliated with microbes^38^. Ultimately, microbes break down river DOM, shuttling compounds between optically distinct components with a net humification of DOM^7^.

In the < 3.0 μm treatment, gradual increases in CDOM and humic-like DOM over the 24-day incubations indicated the potential for larger bacteria to process a broader variety of DOM, including aromatic terrestrial material, either directly or through community effects. The < 0.7 μm treatment fluctuated considerably between time points, suggesting that changing DOM composition and microbial community succession play a large role in production/consumption dynamics for this treatment, with differences in change through time relative to the < 3.0 μm treatment indicating distinct processing.

Microbial transformation of fluorescent components was typically an order of magnitude less than change due to photobleaching. In tandem, however, photobleaching significantly altered trends in microbial transformation and production of fluorophores. CDOM was removed to a greater extent at day 3 and day 24 in the 3.0 μm-after-PB experiment relative to the < 3.0 μm dark treatment. At day 10 in the 3.0 μm-after-PB experiment, CDOM was produced (Fig. 3), suggesting photochemical breakdown of aromatic terrestrial molecules into reactive intermediaries that resulted in a short-term shift in microbial response to diversification of DOM composition, consistent with microbial community succession and a distinct role for larger microbes as stated in our second hypothesis^47^.

The < 0.2 and < 0.7 μm size classes showed limited production and consumption of degraded humics (C4). The < 3.0 μm size class, both in the dark and previously photoexposed treatments, showed significant consumption of C4 from day 0 to day 3. However, after 3 days, two diverging patterns emerged: (1) In the dark treatment, C4 was produced to a greater extent than humics (C1–C3), while (2) previous exposure to light resulted in stable concentrations of C4 after day 3, suggesting either inactivity or static processing. From this, it is likely that relatively complex, photo- and biologically labile molecules contribute to C4 that require functional traits and/or community interactions unique to the < 3.0 μm treatment (present in the 0.7–3.0 μm microbial size class) to degrade in the absence of UV exposure. In the 3.0 μm-after-PB experiment, photolabile compounds that served as precursors to C4 were photochemically altered and unavailable, while in the < 3.0 μm dark treatment, microbial degradation of these compounds produced C4. These results indicate competitive degradation of these precursor compounds by sunlight and microbes, consistent with prior studies of permafrost-derived DOM^13^. Our results show C4 includes functionally heterogeneous fluorophores, including compounds labile to the < 3.0 μm microbial community, photolabile compounds and compounds contributed by photodegradation of HMW terrestrial material. This is consistent with C4 constituting a more degraded component of the DOM pool, evident by C4 fluorescence dominating total fluorescence in oceanic samples. Our results are consistent with production of humics observed by Mann et al.^7^, although our results also highlight how UV exposure can disrupt patterns of processing by altering precursor DOM.

The 3.0 μm-after-PB experiment displayed the largest increase in humics over the 24-day incubation (fluorescence intensity increases of 19.5%, 11.4% and 18.6% for C1, C2 and C3, respectively, relative to initial fluorescence intensity), while all other treatments had a net increase of ≤ 5% (Fig. 4b). As seen in absorption losses, photodegradation enhanced short-term lability, resulting in enhanced DOM consumption and production of humics after an initial drawdown (Table 1). While many studies of fluorescent components attribute C1 and C2 to terrestrial sources, our results show river and coastal ocean microbes increase individual humic contributions by ~ 5% in dark incubations and > 15% in our 3.0 μm-after-PB experiment. C2 was linked to terrestrial material in North Atlantic Deep Water, a water mass sourced from the Arctic Ocean, due to corresponding observations of elevated concentrations of lignin phenols^23,24^. However, C2 has also been linked to in situ production in North Atlantic waters^48^. Our results show that microbial reworking of DOM, especially after exposure to light, can result in humification that is optically similar to fluorescent DOM (peaks A and C) traditionally viewed as terrestrial, in agreement with prior Arctic studies^7^. In this context, elevated concentrations of lignin phenols that still fall below a concentration threshold for microbial use could be observed alongside visible humic material derived from more accessible, potentially non-colored, terrestrial DOM, although the necessity of terrestrial source material is not clear from our dataset^25,49^. Our observations of an increasingly humic signal over time are consistent with observations of successive microbial processing to a diverse, “universal” aquatic DOM pool^50,51^ as well as “universal” fluorescent signatures, consistent with similar functional processing of material by microbes to a convergent optical signature regardless of source^52^. Microbial community structure and function appear to play a role in this process, illustrated by the differences in production of humic fluorescence between size-fractionated inocula and the role of UV exposure in accelerating production of humic material^9,53^. Our results illustrate that optical signatures for DOM carry a microbial processing signature that skews toward humic-like fluorescence that is more distributed in excitation-emission space than precursor DOM, consistent with a DOM pool diversified by microbes, where structurally diverse fluorescent molecules do not directly overlap.

Relative to C1-C4, C5 and C6 were the most variable across treatments and stations (Figs. 4b, S2). These components are typically attributed to protein-like fluorescence (tryptophan and tyrosine-like for C5 and C6, respectively). However, other compounds with similar molecular structures have been nominated as sources of these signals in aquatic environments and are known degradation products from terrestrial vegetation^43,44^. Microbes, including members of the globally abundant SAR202 clade, produce enzymes that break down lignin derivatives and catechols into intermediates used in the Krebs cycle^54^. Catechol, which displays very similar fluorescence to our C5^55^, is a reactionary intermediate in the microbial metabolism of phenols and other compounds^56^, as well as a structural component of siderophores used to acquire trace metals^57^. This diversity of roles lends itself to a number of structurally similar compounds contributing to C5 that would, in turn, exhibit variable behavior over time and across treatments. C5 was initially photoproduced and then subsequently photooxidized in PB experiments, with some stations showing losses of > 20% (Fig. 4b). The 3.0 μm-after-PB experiment showed initial production of C5 from day 0 to day 3, followed by consumption. It appears photodegraded compounds were further metabolized by microbes into C5 at day 3, and these aromatic intermediaries were further degraded for a net removal of C5. Across all microbial experiments, C5 was net removed over time, indicative of wide use of this aromatic material across microbial groups.

C6 was produced across all dark incubation treatments, with moderate consumption occurring for some stations and time points. These trends are in strong contrast to the 3.0 μm-after-PB experiment, which showed that compounds were net consumed. This indicates that photodegradation of complex molecules led to release of molecules with a similar structure to tyrosine. This material was rapidly drawn down and then stabilized, suggesting that photobleaching cleaved larger compounds into small aromatic compounds that were more bioavailable (e.g., dityrosine to tyrosine^40^). Overall, our findings strongly support the hypothesis that photoexposure increases the available pathways for DOM degradation.

### Spatial patterns and fate of freshet DOM in coastal waters

**Figure 5:**
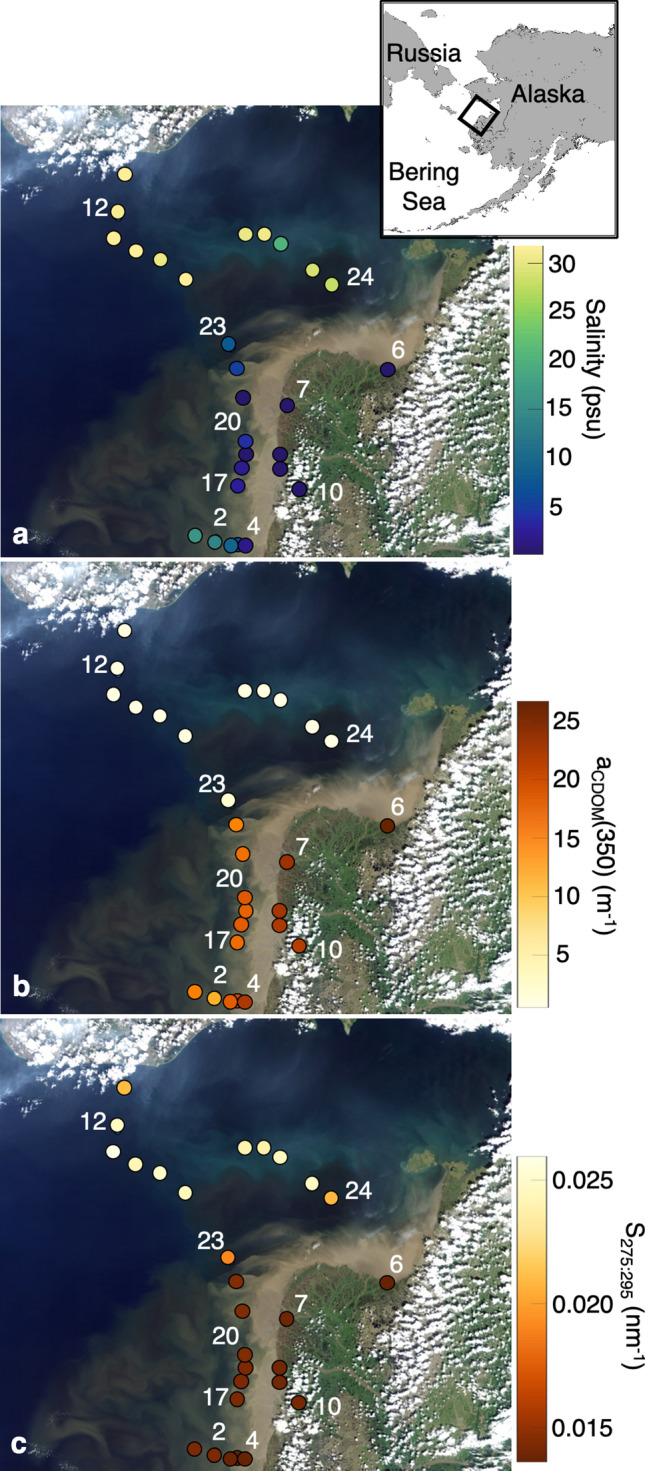
Landsat 8 OLI merged scenes from June 16 and June 18, 2019 of the Yukon River delta and Norton Sound, overlaid with (**a**) salinity, (**b**) a_CDOM_(350) and (**c**) S_275:295_ for all stations sampled (n = 28). Incubation station locations are noted by number. Figure inlay indicates regional location of the Landsat 8 composite.

**Figure 6:**
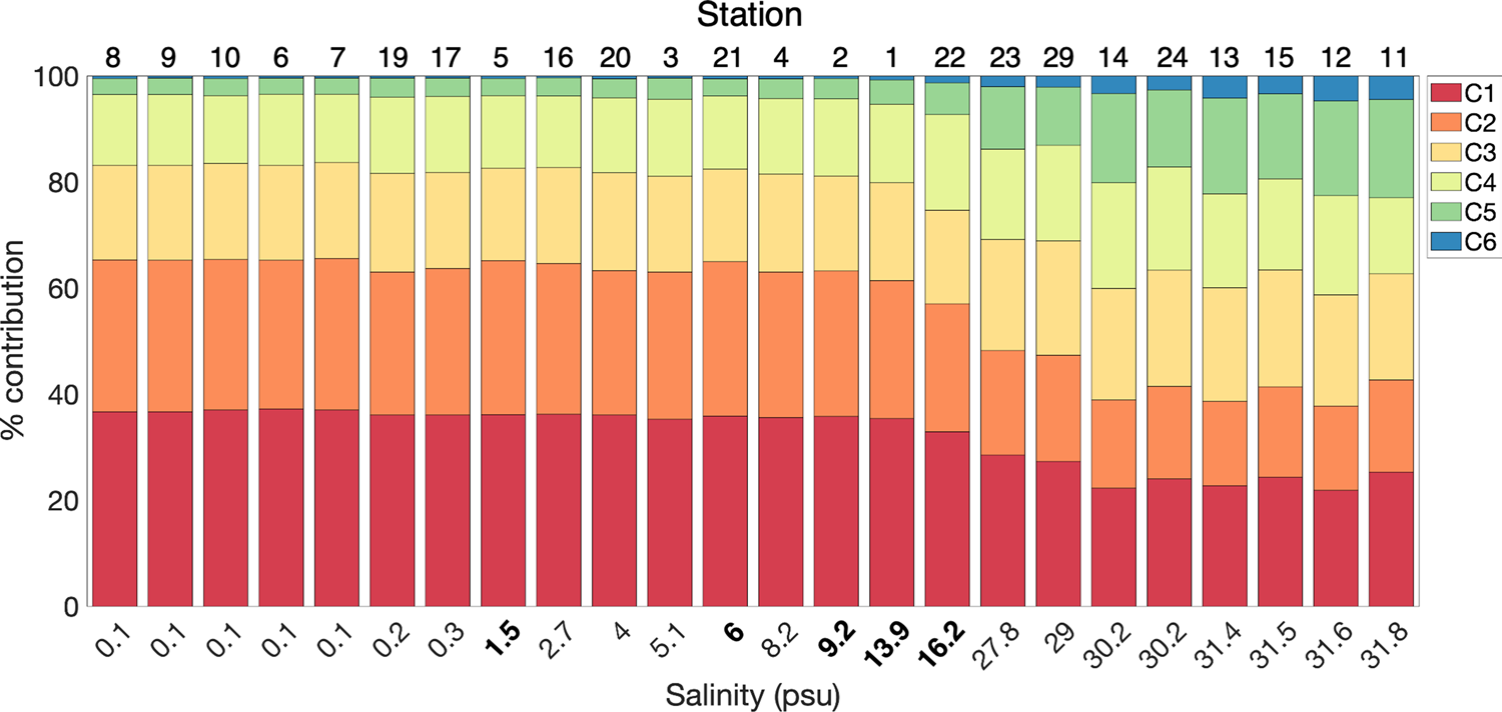
PARAFAC component percent contribution ordered by salinity, with station number indicated above each bar plot. Bolded salinities correspond to stations sampled along a same-day transect of salinity on May 30, 2019, on the southern edge of the Yukon River plume.

Results from our incubation experiments show that Yukon River CDOM is susceptible to rapid photodegradation, with subtle reworking by microbes. Historic observations in the Yukon River, including near our delta sites^41^ and at Pilot Station, AK (~ 200 km upstream) displayed similar spring DOC, a_CDOM_(350) and SUVA_254_ values as those observed here^21,22^. These parameters largely follow established conservative mixing relationships^58^ (Figs. 5, S4). Relative contributions of fluorescent components were mostly stable until higher salinities (~ 16.2 psu), at which point the relative contribution of C1 and C2 decreased and contributions of C5 and C6 increased dramatically (Fig. 6). Relative contributions of C3 and C4 also tended to increase with salinity. Within the plume, solar exposure was quite limited (secchi disk depths ≤ 0.5 m). The change in relative contributions of fluorescent components along the salinity gradient are consistent with our PB experiment results. These results suggest that photochemical processing of CDOM is minimal within the plume while turbidity is high; however, as material is photoexposed, DOM composition is altered in ways that enhance transformation pathways and bioavailability to microbes.

Microbial alteration of DOM subtly shifted within turbid river and plume stations despite apparently conservative relationships between salinity and DOM metrics (Table 1; Fig. S4). Once turbidity decreases, our experimental results show rapid photodegradation of aromatic, terrestrial DOM to biologically labile terrestrial derivatives that were quickly removed by microbes, indicating that the coastal ocean is a hot spot for DOM utilization in the Arctic. Timing of alteration within the plume affected both subsequent photochemical and microbial transformations, showing that timing of exposure to microbes and sunlight can affect net transformation of DOM. Differences in consumption and production of aromatic terrestrial compounds and reactive intermediaries in our incubations, and changes in photochemical response of DOM, indicate subtle shifts in DOM composition through the river and plume (Table 1; Fig. S4). Photochemical losses of C5 were higher in the plume samples (21.8% on average) than in the freshwater river samples (9% on average), with differences in rate not readily accounted for by differences in cumulative exposure between samples (Table S2). In contrast, the < 3.0 μm microbial community removed more DOC from photoexposed media in river samples than in plume samples, consistent with a loss of photochemically and biologically labile terrestrial DOM in plume samples relative to river samples. River samples displayed more production of C6 than plume samples in the PB experiment, and also displayed higher consumption rates of C6 than plume samples in the 3.0 μm-after-PB experiment. These findings all support microbial alteration of this material through the river-ocean continuum and a microbial response tailored to changing DOM composition.

Overall losses in DOC were not significantly different between microbial processing of photoexposed and non-photoexposed DOM. Thus, while photochemistry alters the timing of DOM use by specific microbial groups, including removal of biologically and photo-labile compounds, it does not necessarily alter net DOC remineralization, contrary to our hypothesis. Our results showed up to 20% loss of DOC during microbial processing, with the highest utilization of river and plume DOC by the < 3.0 μm microbial community, relative to < 6% change in a_CDOM_(300). Sunlight strongly regulates DOM composition and timing of consumption for specific compounds; however, microbes play a critical role in DOM remineralization, with higher DOC remineralization rates by the < 3.0 μm microbial community and in river samples. Prior work has indicated that larger microbes are more productive and more abundant in samples enriched with terrestrial DOM^26,59^; trends in consumption of C4 observed in our < 3.0 μm treatments support a unique role for larger microbes either through direct processing and/or community effects. Notably, our results show compositional shifts in DOM that suggest bioavailability is linked to microbial functional traits unique to the inclusion of the 0.7–3.0 μm microbial community, which leads to higher carbon remineralization rates. Thus, our results indicate large microbes play a key role in degradation of terrestrial material in coastal Arctic waters, including competition with photochemical transformation pathways^60,61^, and point towards new areas of research to further elucidate the role of microbial size. A deeper understanding of how environmental conditions and changing DOM composition impact microbial community structure and function, along with knowledge on specific degradation pathways of photosensitive aromatic compounds, will help address sensitivity of freshet DOM to remineralization in coastal Arctic waters, and residence time within the global carbon cycle.

## Methods

### Study site

Sampling of the Yukon River delta, plume and adjacent coastal waters was conducted as part of a larger Arctic remote sensing of water quality field campaign, with aims of capturing seasonal and spatial biogeochemical variability along a large Arctic river-coastal ocean salinity gradient. 28 stations were sampled immediately after spring freshet and during peak Yukon River discharge, to assess in situ conditions. Of these 28 stations, ten were selected to assess the impact of sunlight and microbes on DOM processing across the salinity gradient (Fig. S1). Stations were chosen based on location along a direct salinity gradient (Stations 2, 4 10, salinity = 0.1–13.9 psu), within the Yukon delta and major river mouths (Stations 6, 7 and 10, salinity = 0.1 psu), across anticipated within-plume salinity-induced flocculation gradients (Stations 2, 4, 17, 20 and 23, salinity = 2.7–13.9 psu), immediately outside the plume (Station 24, salinity = 27.8 psu) and a high salinity, oceanic end-member station (Station 12, salinity = 31.2 psu). These samples were collected on two separate legs of the spring freshet field campaign: Yukon delta sampling was conducted out of a small vessel (~ 20 ft), based from Alakanuk, AK from May 30 to June 3, 2019 and Yukon plume and Norton Sound sampling conducted out of Nome, AK aboard a coastal class vessel (~ 70 ft) from June 9 to 11, 2019.

### Sample collection

Temperature, salinity, dissolved oxygen, turbidity, chlorophyll and DOM fluorescence for each station were determined immediately using a YSI ExO2 Sonde and used as the basis for determining which stations to sample for lab incubations. Water was acquired using either a bottle grab or a peristaltic pump sampling within 1 m of the water surface, with sample bottles pre-cleaned and triple rinsed with sample water prior to collection. Samples were filtered using a Geotech 0.45 μm Medium Capacity dispos-a-filter (non-woven substrate) either in the field or in the lab, within 10 h of sampling, and stored in amber glass bottles for CDOM/DOC analysis or 2L brown plastic Nalgene bottles for incubation media until further processing (see “Photobleaching and microbial incubations”). A total of 4 L of water was collected at each station for incubation experiments. Inocula used for microbial degradation experiments were filtered on station through either a 0.7 μm or 3.0 μm nylon syringe filter into a sterile 125 mL brown plastic Nalgene bottle, pre-rinsed three times with sample. Water samples (including inocula) were stored at 4 °C while on site in Alakanuk and Nome. All samples were shipped and received within 48 h, with sample analysis occurring within 1–3 weeks of sampling. All incubation experiments began within 1 month of sampling.

### Photobleaching and microbial incubations

Incubation media initially filtered through 0.45 μm (see “Sample collection” above) were filtered again through an 0.22 μm PES capsule filter within 24 and 10 days of storage (Alakanuk and Nome, respectively). There were three distinct incubation experiments: (1) 24-day photobleaching experiments of 0.22 μm filtrate (herein referred to as "PB"); (2) 24-day dark incubations of 0.22 μm filtrate incubated with no inoculum added, < 0.7 μm and < 3.0 μm inoculum sampled from the respective station (herein referred to as 0.2, 0.7 and 3.0 μm treatments, respectively, or MD dark experiments collectively); and (3) 24-day dark incubation of 0.22 μm filtrate that was previously exposed to light (for 7 days) and incubated with < 3.0 μm inoculum sampled from the respective station (herein referred to as "3.0 μm-after-PB"). Size-fractionation was used as a simplified proxy of microbial capability to degrade high molecular weight, aromatic terrestrial DOM. We assumed a gradient effect, where some microbes present in the < 0.7 μm fraction would grow to become larger than this initial size threshold. However, within this approach, we also assumed that there would be microbes that even at their smallest size would still be larger than the < 0.7 μm threshold, and thus would be uniquely present in only the < 3.0 μm inoculum throughout the course of the experiment^62^.

Incubation experiments began with a 7 day photoexposure experiment run to acquire the 7 day timepoint used in all photobleaching results, and to provide photoexposed media for the 3.0 μm-after-PB experiment. For the photoexposure experiment, 160 mL of 0.22 μm filtrate was stored in a 250 mL Teflon (FEP) bottle, incubated in duplicate. This resulted in 20 bottles (2 per station). The 0.22 μm filtrate was exposed to UV light from 280 to 400 nm from the bottom of the bottle, through transmitting UVT Plexiglas (Fig. S5). This experimental setup allowed for > 80% of light to pass through the Plexiglas sheet, with measured UV exposure in the lab approximately 60% of that observed in the field, representing values approximately equivalent to average in situ exposure in the first optical depth (see “Supplementary Methods”). Media was photoexposed for 7 days (168 ± 1 h). Bottles were evenly spaced along the length of each fluorescent bulb in two rows (A and B duplicate rows) initially. Samples were mixed (inverted twice) and bottles were rotated daily to ensure approximately uniform incident light exposure across bottles, as light intensity along the length of each fluorescent light bulb varied up to 11% at the ends of the bulbs (< 2% variability along the middle 8 bottles; Fig. S5). After sub-sampling this media for optical analysis, the remaining media was used for the photobleaching and microbial degradation (3.0 μm-after-PB) experiment.

During this initial 7-day period, 160 mL of 0.22 μm filtrate used for the PB (only light) treatment was stored for 1 week at 4 °C in duplicate 250-mL Teflon bottles for each station, and then exposed to light for 24 days, following the same light-exposure protocol as above. PB samples were measured for change at 1, 3, 14 and 24 days, with the 7 day timepoint provided from the first photoincubation experiment. 35 mL of water was removed from the Teflon bottle for absorption and fluorescence at each timepoint without re-filtering prior to analysis, with DOC additionally measured at the 24 day timepoint.

To examine microbial degradation, we conducted 24-day incubations of photoexposed DOM with the < 3.0 μm inoculum, as well as non-photoexposed DOM with < 0.7 and < 3.0 μm inocula. These incubations began after the initial 7-day period (samples stored at 4 °C), to allow for all 24-day incubations to occur coincidentally. Six bottles, per station, were used: (1) four 480 mL amber glass bottles with 400 mL of non-exposed 0.22 μm filtrate, two of them inoculated with 1 mL of < 0.7 μm microbial inoculum and two inoculated with 1 mL of < 3.0 μm microbial inoculum (“0.7 μm” and “3.0 μm” treatments, respectively), (2) one 480 mL amber glass bottle with 200 mL of previously photoexposed 0.22 μm filtrate, inoculated with 0.5 mL of < 3.0 μm microbial inoculum and (3) one 250 mL clear glass bottle with 200 mL of control incubation media (i.e., non-photoexposed 0.22 μm filtrate) without microbial inoculum addition, used as the experimental control. All incubations were conducted at 10 °C to mimic average temperatures observed in situ (~ 9–11 °C for all river and plume stations, and 8.0 °C at St 12). Bottles were aerated and mixed daily by briefly removing the cap (< 10 s), then closing and inverting the bottle twice. MD and 3.0 μm-after-PB samples were measured for optical change at 3, 10 and 24 days, and change in DOC at 24 days. 60 mL of water was removed from sample bottles at each timepoint and the water was filtered through a 0.22 μm disc filter prior to analysis for all samples, including the control.

Samples for analyzing microbial cell counts and biomass were collected on all dark incubations, with 5 mL of sample extracted from each bottle at 1, 3, 10 and 24 days. Samples were preserved with 2% glutaradlehyde and stored at 4 °C until analysis on a Guava easyCyte flow cytometer. Prior to sample collection, a shared use Guava easyCyte 5 flow cytometer was identified for use; however, upon return from the field, the instrument was unexpectedly at sea. Upon instrument return, initial samples collected were approximately 6 weeks old. 50 μL of sample was stained with SYBR Green I dye and cell counts were reported within the InCyte software, based on detection of green DNA fluorescence. As noted in the literature, the quality of the samples were likely comprised and cell counts were deemed unreliable^63^. Nevertheless, sample quality was deemed sufficient to indicate the presence or absence of bacterial cells. All control bottles (0.2 μm filtrate) displayed evidence of significant microbial biomass (cell counts an order of magnitude greater than SYBR-stained deionized water controls), as found in prior studies^64^. From this, we considered the control bottles as a 0.2 μm MD treatment. This also indicated that some degree of microbial activity was present in all PB experiments consistent with rapid growth of bacteria even under UV exposure in a previous study using the same incubation design^64^. It is noted that the microbes present in the < 0.2 μm filtrate are also expected to be present in the < 0.7 and < 3.0 μm microbial inocula (Fig. S6).

### Optical and chemical analysis

CDOM absorption was measured using a Cary 300 dual-beam spectrophotometer in a 5 cm pathlength quartz cuvette^65^. Cuvettes were cleaned before analysis and rinsed with MilliQ then sample water between samples. Measurements were baseline-corrected using MilliQ water, and MilliQ blanks were subtracted from individual scans. Triplicate measurements were performed for each sample, with individual scans assessed as outliers and all quality-controlled scans then used to estimate an average absorbance for the sample^66^. Absorption was measured from 220 to 750 nm (1-nm bandwidth and interval). Absorbance values were converted to CDOM (Napierian) absorption coefficients (a_CDOM_(λ)) following:

1 $${a}_{CDOM}(\lambda )=2.303\frac{A(\lambda )}{l}$$

where A(λ) is the measured absorbance and *l* is the pathlength in meters (here, 0.05)^67^. Once converted to absorption, spectra were modeled using a nonlinear fitting routine to compute spectral slope from 275–295 and 350–400 nm (S_275:295_ and S_350:400_, respectively):

2 $${a}_{CDOM}\left(\lambda \right)={a}_{CDOM}({\lambda }_{0}){e}^{-S(\lambda -{\lambda }_{0})}$$

where λ is wavelength (nm), the reference wavelength (λ_0_) is the average wavelength for the spectral range being fit (285 or 375 nm) and S is the spectral slope (nm^−1^)^66^. Dissolved organic carbon was measured on a Shimadzu TOC-V Total Organic Carbon analyzer. A few samples exhibited larger DOC values at day 24 than at day 0 and were excluded from analysis (e.g., St 12 in the 0.2 μm treatment, Table 1). The specific UV absorbance at 254 nm (SUVA_254_) values were determined by dividing CDOM absorbance at 254 nm by measured DOC concentration and are reported in units of liter per milligram carbon per meter^68^.

CDOM fluorescence was measured on a Horiba Aqualog Spectrofluorometer (Jobin Yvon Horiba) using a 1-cm quartz cuvette. On each day of sample analysis, a reference was run for quinine sulfate fluorescence and a blank was run using a sterile MilliQ water cuvette. Samples were analyzed at 5 nm increments of excitation from 240 to 650 nm with an integration time of 1 s. Excitation emission matrices (EEMs) from the Aqualog represented a ratio of emission detector output to that of the reference detector (S/R). Within the Aqualog software, both the reference signal and emission detector signal had dark signals subtracted and spectral corrections applied. EEMs outputted from the Aqualog were loaded into a Matlab structure and aligned for input into the drEEM toolbox, where corrections were made for the inner filter effect based on absorbance coefficients of aligned samples run on the Cary spectrophotometer. Sample EEMs had fluorescence signal from MilliQ water subtracted, based on blank EEMs that had been run on the same day. These MilliQ water EEMs were also used to calculate Raman peaks, to then convert sample EEMs to fluorescence in Raman Units.

### PARAFAC

An initial EEMs dataset including all incubation DOM samples (n = 285), other DOM samples from the Yukon River and Norton Sound sampling in 2019 (n = 26) and DOM samples from the Elson Lagoon and local freshwater systems near Barrow, AK (n = 28) were run through the exploratory data analysis functions within the drEEM toolbox to identify outliers and the appropriate number of fluorescent components to model using Parallel Factor Analysis (PARAFAC)^69–71^. Of the initial 339 EEMs, 33 were removed as outliers, including 26 incubation EEMs accounted for primarily by Station 12 samples (highest salinity and lowest fluorescent signal). A six component model that explained > 99.9% of total variation was identified as the best fit using split half analysis to validate the model (Fig. S7). All components co-varied independently. PARAFAC modeling of EEMs from all field and incubation samples (n = 306) produced six components (C1-C6), with St. 12 excluded from analysis due to many EEMs removed for PARAFAC validation. We published our modeled components to the OpenFluor database^44^ and compared to published components on OpenFluor.

Percent change in absorption and fluorescent components over time, in Raman units (R.U.), was calculated as

3 $$\Delta C\left(x\right)=100 \cdot \left(\frac{{C(x)}_{i+t}-{C(x)}_{i}}{{C(x)}_{i}}\right)$$

where *C(x)*_*i*_ represents the initial fluorescent component loading (day 0), *C(x)*_*i*+*t*_ represents the fluorescent component loading at time *t* (days), and *ΔC(x)* is percent change in PARAFAC component at time *t*. Percent changes were related to the initial component fluorescence to ensure percent changes were comparable across time points. Component changes largely followed a normal distribution across sites and time points; thus, outliers were identified using Grubb’s test for outliers, which removes one outlier per iteration based on a failure to adhere to a normal distribution. In practice, this removed any component changes greater than ± 80% between individual timepoints.

Percent contribution of each component to total fluorescence was calculated as the fluorescence maxima (component loading in R.U.) divided by the sum of fluorescence maxima for all components, e.g.,

4 $${C}_{i} \%=\frac{{C}_{i} {F}_{max}}{{\sum }_{i=1}^{6}{C}_{i} {F}_{max}}*100$$

where C_i_ represents one of the six fitted components and F_max_ is derived from the component loading output of PARAFAC.

## Supplementary Information

Supplementary Information.
