# Supplementary material for: DOM degradation by light and microbes along the Yukon River-coastal ocean continuum
Source: Sci Rep. 2021 May 13;11:10236. doi: 10.1038/s41598-021-89327-9 (PMC8119953; doi:10.1038/s41598-021-89327-9)
Supplement: Supplementary file 1 — Supplementary Information. [file 41598_2021_89327_MOESM1_ESM.docx]

**Supplementary Material for**

**DOM degradation by light and microbes along the Yukon River-coastal ocean continuum** Brice K. Grunert^1*^, Maria Tzortziou^1^, Patrick Neale^2^, Alana Menendez^1^, Peter Hernes^3^

^1^The City College of New York, The City University of New York, Department of Earth and Atmospheric Sciences, 160 Convent Avenue, New York, New York 10031

^2^Smithsonian Environmental Research Center, 647 Contees Wharf Rd, Edgewater, MD, USA 21037

^3^University of California, Davis, Department of Land, Air, and Water Resources, Davis, CA, USA 95616

^*^Corresponding author: bgrunert@ccny.cuny.edu

**Supplementary Methods**

**Irradiance Calculations**

*1. Incubation Irradiance*

Base spectrum (*E*(λ), mW m^-2^ nm^-1^) is that measured for the Logozzo et al. incubations at 20°C with associated in air PAR measurements at the location of each bottle (above Plexiglas sheet, see Fig. S1-S4)^1^. This was adjusted by the ratio of PAR measurements made here for each volume of water inside the bottle relative to the Logozzo et al. in air PAR values^1^. Bottle PAR was measured with a scalar (4π) irradiance probe (Biospherical QSL2100 with immersion correction) centered in DI of volume corresponding to the sample volume of each incubation. The ratio of bottle PAR to air PAR was taken as a correction factor for refraction and scattering by the curved surfaces of the Teflon (FEP) container. Applying this factor to the base spectrum from Logozzo et al. and also applying the spectral UV transmission of Teflon, we obtained the average UV spectrum, *E*(λ) (DI only), for each incubation^1^. The UV transmittance was normalized to PAR transmittance at 450 nm (since the PAR measurement inside the bottle includes the Teflon transmittance). In general, the average *E*(λ) spectra were about half of the Logozzo et al. exposures, chiefly due to a lower voltage output from the power supply, with secondary effects due to lower temperatures used here (10°C) impacting light output of the fluorescent bulbs^1^. Average UV irradiance (284-400 nm) at the bottom of the bottle was 5.6 W m^-2^.

To account for shading effects of CDOM, spectral correction factors were estimated by assuming that irradiance inside the bottle was approximately collimated over a vertical pathlength, *l* (m):

$Average E\left( \lambda\right)=Average E\left( \lambda\right) \left( DI only \right)*(1-e^{(-abs\left( \lambda\right)*l)/(abs\left( \lambda\right)*l))})$ (S1)

where abs(λ) is the absorption of the sample (m^-1^) measured on a spectrophotometer. Average total UV exposure inside the bottle was then the integral of E(λ) from 284-400 nm. The effective absorption for each exposure interval was taken as the geometric mean of absorption at the beginning and end of each interval.

Pathlength was estimated by assuming it was proportional to volume, based on a measured vertical pathlength of 4 cm for a volume of 100 mL. Based on that proportion, 160 mL =6.4 cm, 125 mL = 5cm, 90 mL = 3.6 cm, 55 mL = 2.2 cm.

*2. Incubation Cumulative Exposure*

Cumulative UV dose for each treatment was calculated as the product of Average UV and time for each interval. The incubation volumes during the intervals were as follows:

T0 to T1 – 160 ml

T1 to T3 – 125 mL

T0 to T7 – 160 mL

T3 to T14 – 90 mL

T14 to T24 – 55 mL

Using the average irradiance for each of these intervals estimated using Equation S1, a cumulative exposure (MJ/m^2^) was calculated for each PB sample point by multiplying the irradiance (W/m^2^) by interval duration in s (Table S2).

*3. Comparison to In Situ Surface Ocean Exposure*

Using the date, time and location of sampled stations, the radiative transfer models SMARTS295 and QuickTUV were used to estimate clear sky data to coincide with in situ incident irradiance data collected by a Satlantic HyperOCR reference sensor mounted on the research vessel to avoid any shading. A moderate ozone value of 300 DU and low aerosol optical density was used in the model runs. In the SMARTS295 runs, a triangular filter function was applied to the fine resolution (0.5 nm) model output to approximate the bandwidth of the HyperOCR optics (FWHM = 10 nm). An example comparison of the SMARTS295 and Satlantic HyperOCR spectra for Station 10 is presented in Fig. S8.

The shape of the Satlantic HyperOCR spectrum follows the clear sky at about 67% proportion (interpreted as cloudiness effect) down to 350 nm. Below that wavelength, the Satlantic HyperOCR spectrum appears to fall off more sharply than the model spectrum, consistent with reported wavelength fidelity between 350-800 nm and less reliable data below that wavelength range by Sea-Bird Scientific. This was a consistent feature for all stations considered (n=10) and was also seen in comparison of the Satlantic HyperOCR spectrum and QuickTUV output. From this, it was determined that model output was reliable and accurate for clear sky conditions. These models have also been extensively validated by comparison to spectroradiometer measurements in other locations^2^. A similar model clear sky vs. measurements comparison was made for the USDA multichannel radiometer in Fairbanks, AK (64.84°N) averaging data from May 31 to June 11, 2019.

The average ratio of observed to clear sky irradiance over the range of 350-400 nm (0.6546) was used as the “cloudiness factor” for the Yukon cruise period. This is a smaller proportion of clear sky than calculated for Fairbanks (0.7435), which is consistent with the expectation of more cloudiness open water. In both cases, the cloudiness factor was used to adjust SMARTS295 model output from 290-400 nm calculated at hourly intervals over the full daylight period to estimate daily incident UV. The increased cloudiness decreases the daily UV exposure from the Fairbanks estimate of 1.24 MJ m^-2^ d^-1^ to incident Yukon of 1.12 MJ m^-2^ d^-1^. Applying a further correction for surface albedo (Fresnel correction applied to direct beam) brings the estimate of average daily exposure “just below surface” assuming “average cloudiness” down to 1.05 MJ m^-2^ d^-1^. The incubation daily UV exposure (not accounting for the self-shading effects of CDOM) ranges from 61-69% of this exposure depending on volume (average 63%). Based on this estimate, we infer that the incubation exposures are comparable to the average UV exposure (284-400 nm) in the first optical depth, i.e., 1- *e*^-1^ = 0.63.

**Supplementary Tables**

Table S1. Photobleaching relationships for a_CDOM_(300) versus cumulative UV exposure versus time.

| Station | Equation | Coefficients | r^2^ | n |
| --- | --- | --- | --- | --- |
| St 2 | f(x) = a⋅e^(b*x)^ + c⋅e^(d*x)^ | a = 24.0163, b = -0.0648, c = 2.6830, d = -1.7464 | 0.99 | 12 |
| St 4 | f(x) = a⋅e^(b*x)^ + c⋅e^(d*x)^ | a = 37.1452, b = -0.0748, c = 2.6847, d = -1.6351 | 0.99 | 12 |
| St 6 | f(x) = a⋅e^(b*x)^ + c⋅e^(d*x)^ | a = 48.0342, b = -0.0583, c = 3.5602, d = -2.069 | 0.99 | 12 |
| St 7 | f(x) = a⋅e^(b*x)^ + c⋅e^(d*x)^ | a = 47.0897, b = -0.0626, c = 3.6449, d = -2.4029 | 0.99 | 12 |
| St 10 | f(x) = a⋅e^(b*x)^ | a = 49.1490, b = -0.0674 | 0.99 | 12 |
| St 12 | f(x) = a⋅e^(b*x)^ | a = 1.5365, b = -0.0495 | 0.99 | 12 |
| St 17 | f(x) = a⋅e^(b*x)^ + c⋅e^(d*x)^ | a = 34.48, b = -0.0701, c = 3.0444, d = -1.9446 | 0.99 | 12 |
| St 20 | f(x) = a⋅e^(b*x)^ + c⋅e^(d*x)^ | a = 36.2459, b = -0.0616, c = 3.4627, d = -1.956 | 0.99 | 12 |
| St 23 | f(x) = a⋅e^(b*x)^ + c⋅e^(d*x)^ | a = 30.6556, b = -0.0617, c = 3.4892, d = -1.6104 | 0.99 | 12 |
| St 24 | f(x) = a⋅e^(b*x)^ | a = 4.1805, b = -0.0634 | 0.99 | 12 |

Table S2. Cumulative dose (MJ/m^2^) in the photobleaching experiment for each station and timepoint.

| Day | St 2 | St 4 | St 6 | St 7 | St 10 | St 12 | St 17 | St 20 | St 23 | St 24 |
| --- | --- | --- | --- | --- | --- | --- | --- | --- | --- | --- |
| 0 | 0 | 0 | 0 | 0 | 0 | 0 | 0 | 0 | 0 | 0 |
| 1 | 0.437 | 0.368 | 0.322 | 0.326 | 0.323 | 0.622 | 0.381 | 0.373 | 0.399 | 0.598 |
| 3 | 1.404 | 1.211 | 1.073 | 1.087 | 1.076 | 1.870 | 1.250 | 1.226 | 1.302 | 1.812 |
| 7 | 3.195 | 2.728 | 2.363 | 2.399 | 2.385 | 4.360 | 2.818 | 2.743 | 2.931 | 4.184 |
| 14 | 7.412 | 6.769 | 6.126 | 6.209 | 6.162 | 8.770 | 6.884 | 6.762 | 7.054 | 8.616 |
| 24 | 13.386 | 12.588 | 11.614 | 11.755 | 11.672 | 15.079 | 12.720 | 12.513 | 12.905 | 14.893 |

**Supplementary Figures**

**
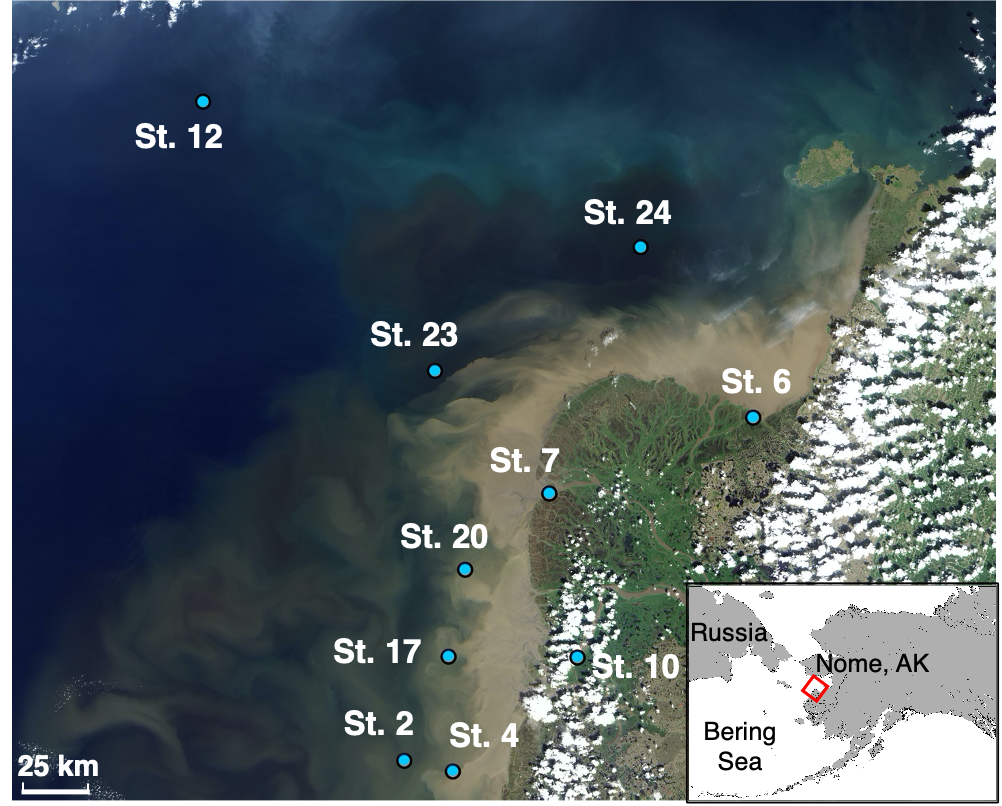
**

Figure S1. Landsat 8 OLI merged scenes from June 16 and June 18, 2019 of the Yukon River delta and Norton Sound, corresponding to the red box noted on the inlaid map. Incubation station locations are shown in blue circles. Stations 6, 7 and 10 were sampled at the northern, middle and southern (main) mouths of the Yukon River, Stations 2, 4, 17, 20, 23 and 24 were within the Yukon River plume at the time of sampling and Stations 12 was outside the plume.

Salinity (psu)

**
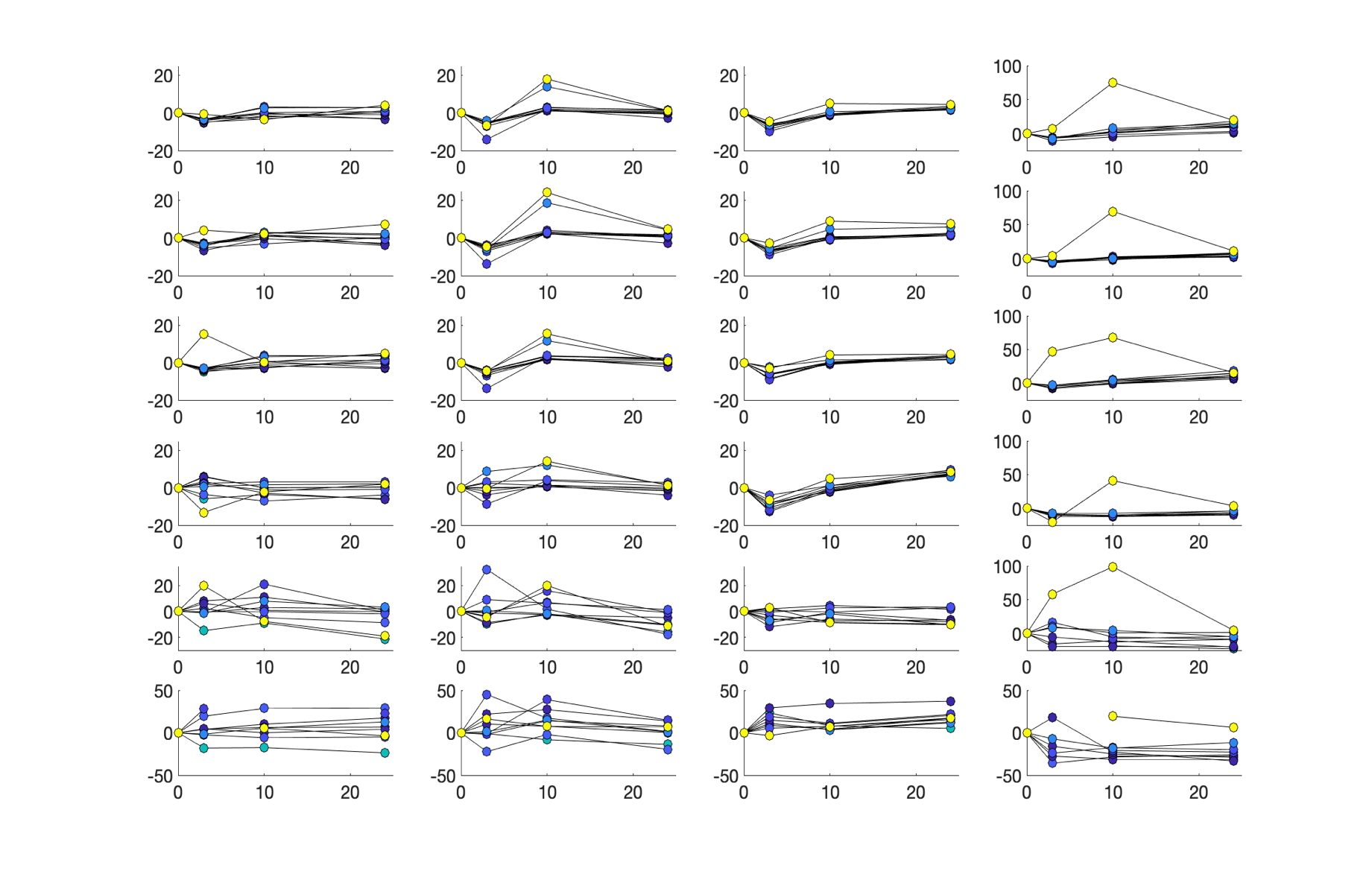
**

Days

ΔC (%)

C6

C5

C4

C3

C2

C1

3.0 μm after PB

3.0 μm

0.7 μm

0.2 μm

Figure S2. Response of modeled PARAFAC components over time, across the different microbial treatments. All stations except St. 12 are included, with salinity denoted by marker color. Overall response to treatment is consistent across salinities, except for the 3.0 μm after PB treatment, where St. 24 often departed from overall trends at days 3 and 10.

Emission (nm)

Excitation (nm)

Figure S3. PARAFAC component fingerprints with more detailed excitation and emission axes than shown in the manuscript.


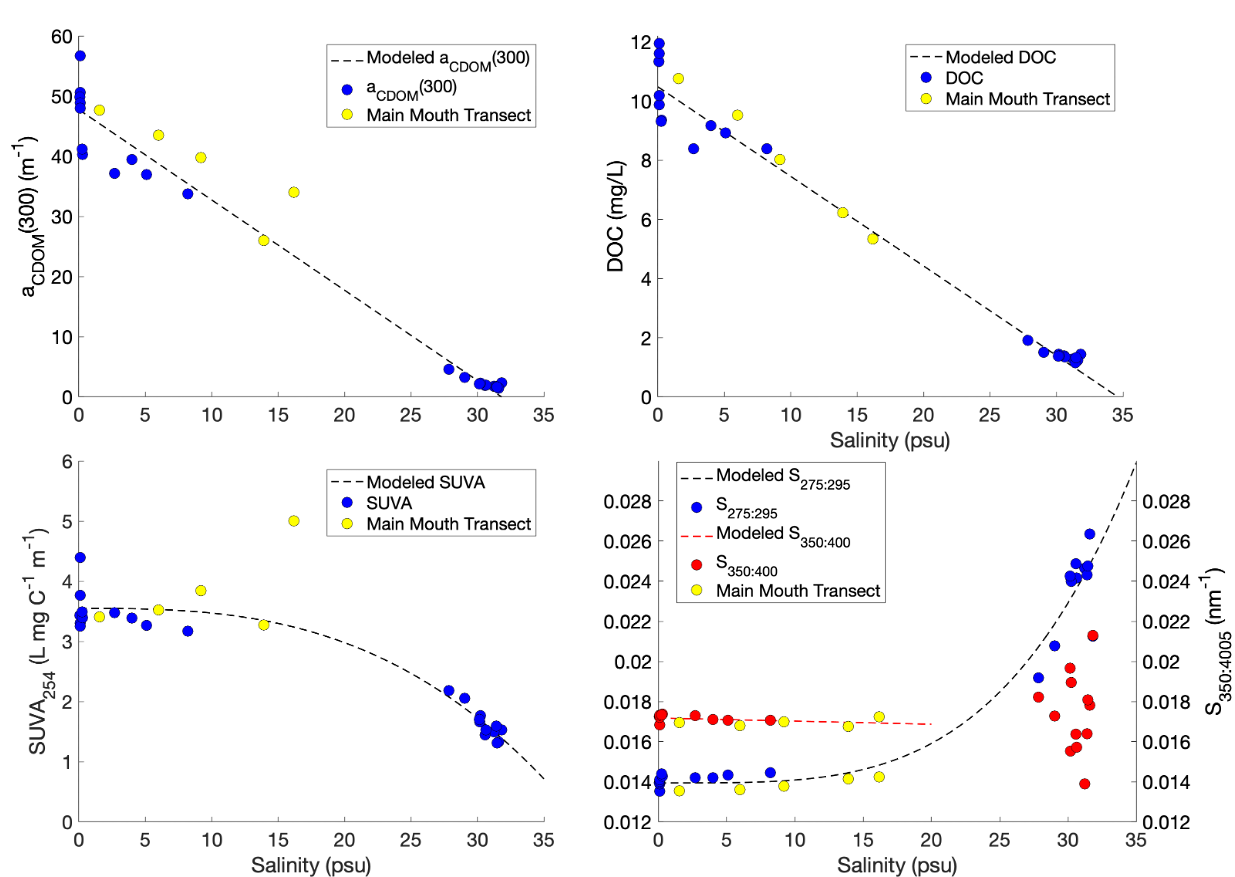


Figure S4. Relationships between salinity and (a) CDOM absorption at 300 nm, (b) dissolved organic carbon, (c) specific UV absorbance at 254 nm and (d) spectral slope of CDOM absorption from 275-295 and 350-400 nm. The dashed lines in all figures represent modeled fits between salinity and each variable following a (a) linear (r^2^=0.94) , (b) linear (r^2^=0.96), (c) second order power law (r^2^=0.78) and (d) second order power law relationship (r^2^=0.96), with the red dashed line in (d) representing the trend line for S_350:400_ for salinities below <20 psu. In all figures, yellow circles represent a single day transect along the salinity gradient on the southern edge of the Yukon River plume, approximately following the direction of discharge from the main stem (south mouth) of the Yukon River. All relationships followed behavior expected for conservative mixing (Stedmon and Markager 2003).


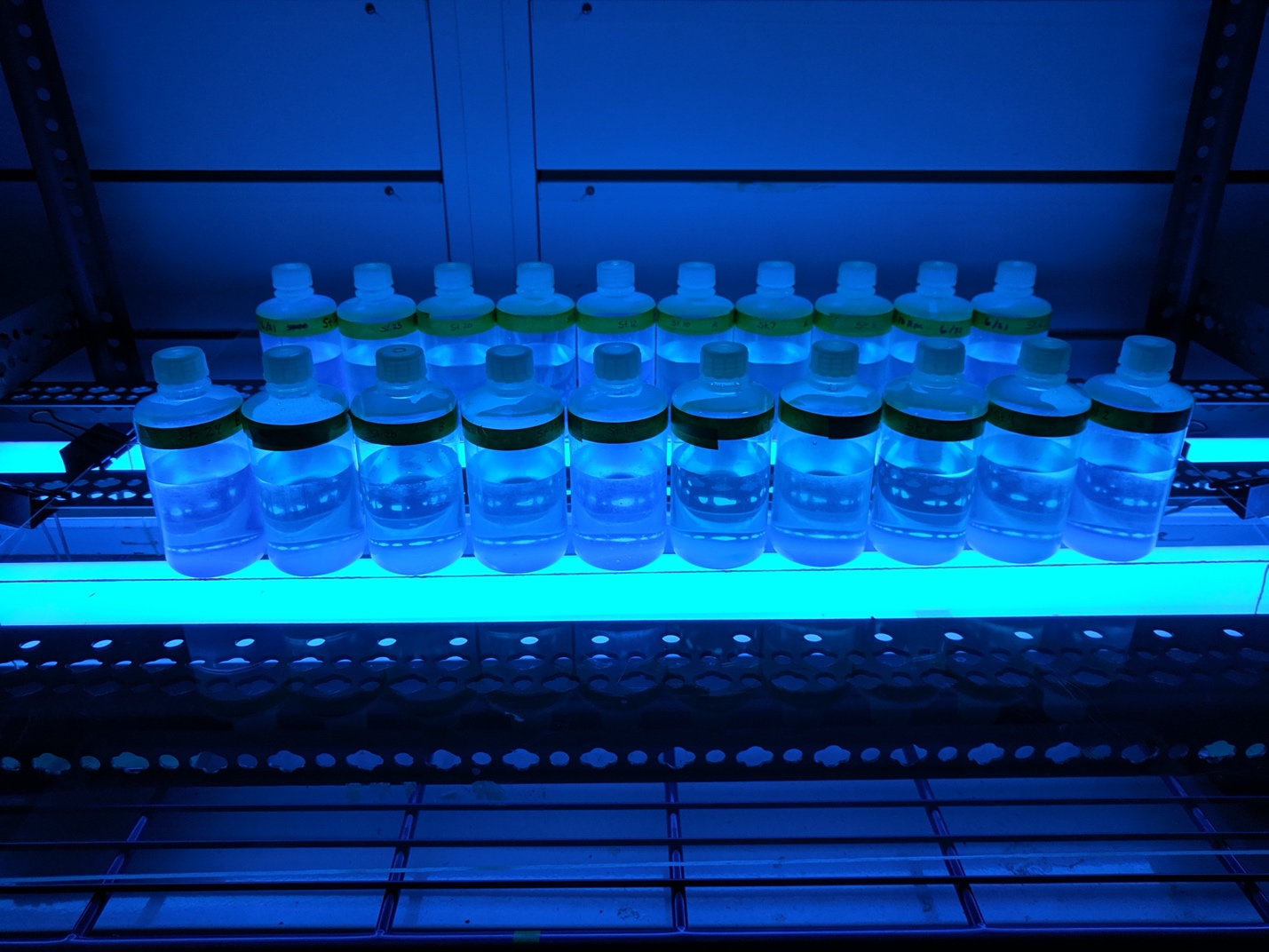


a

b


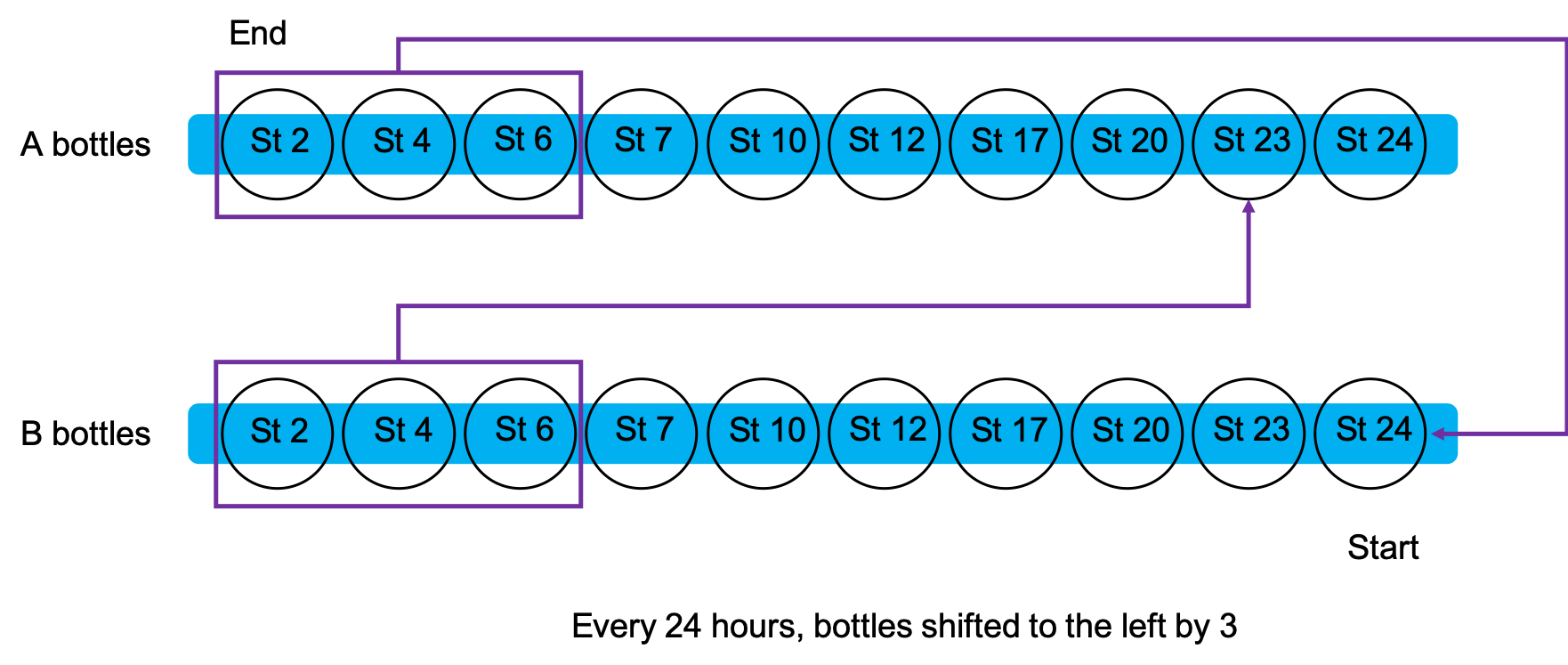


Figure S5. (a) Photo of photobleaching setup and (b) schematic depicting initial bottle locations and rotation pattern. The blue cylinder indicates the location of the fluorescent bulb, which was located directly underneath the bottles, separated by the UV transparent Plexiglas sheet. Scalar irradiance within the bottles was measured with a 4π QSL probe to characterize the irradiance field within each bottle.


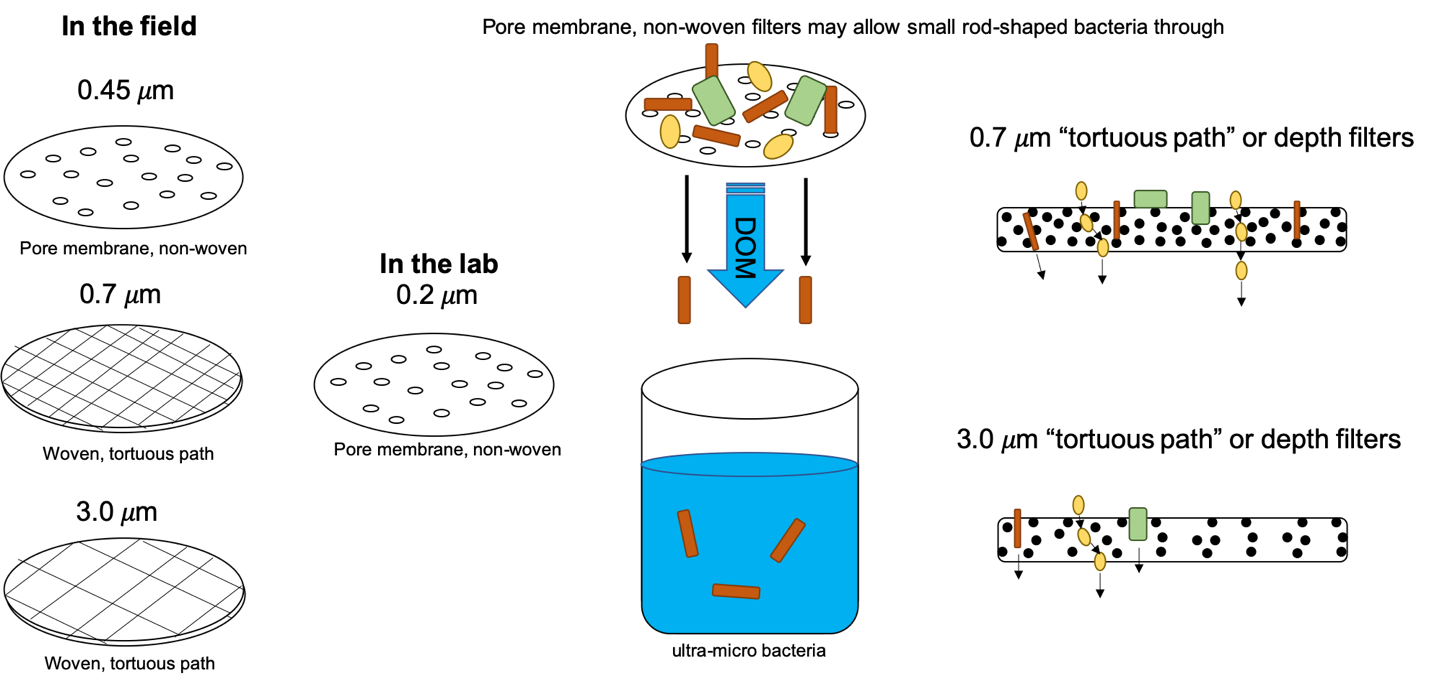


Figure S6. Schematic depicting types and pore/mesh size of different filters used as part of this study and potential for “ultra-micro bacteria” to pass through pore membrane, non-woven filters. This phenomenon has been noted in prior studies, although passage through a filter is assumed and not shown in this study or other studies noted in the paper.

**
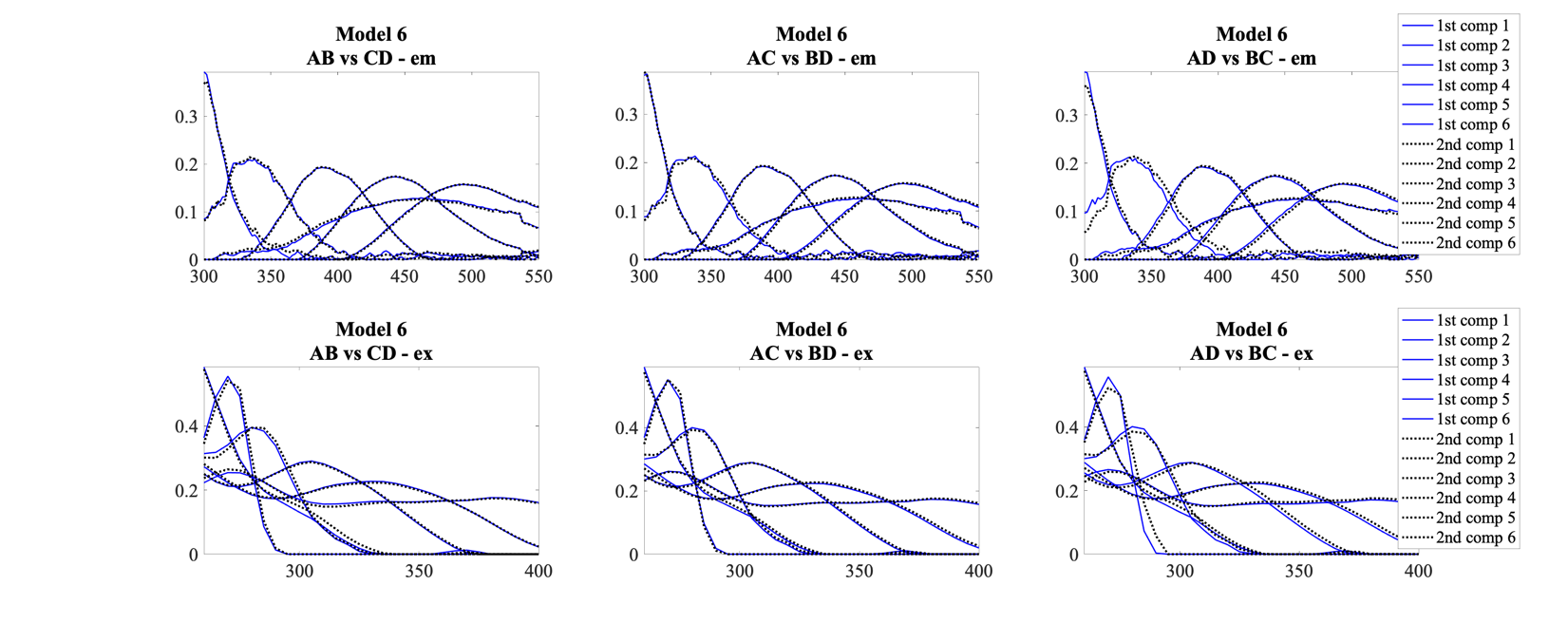
**

**
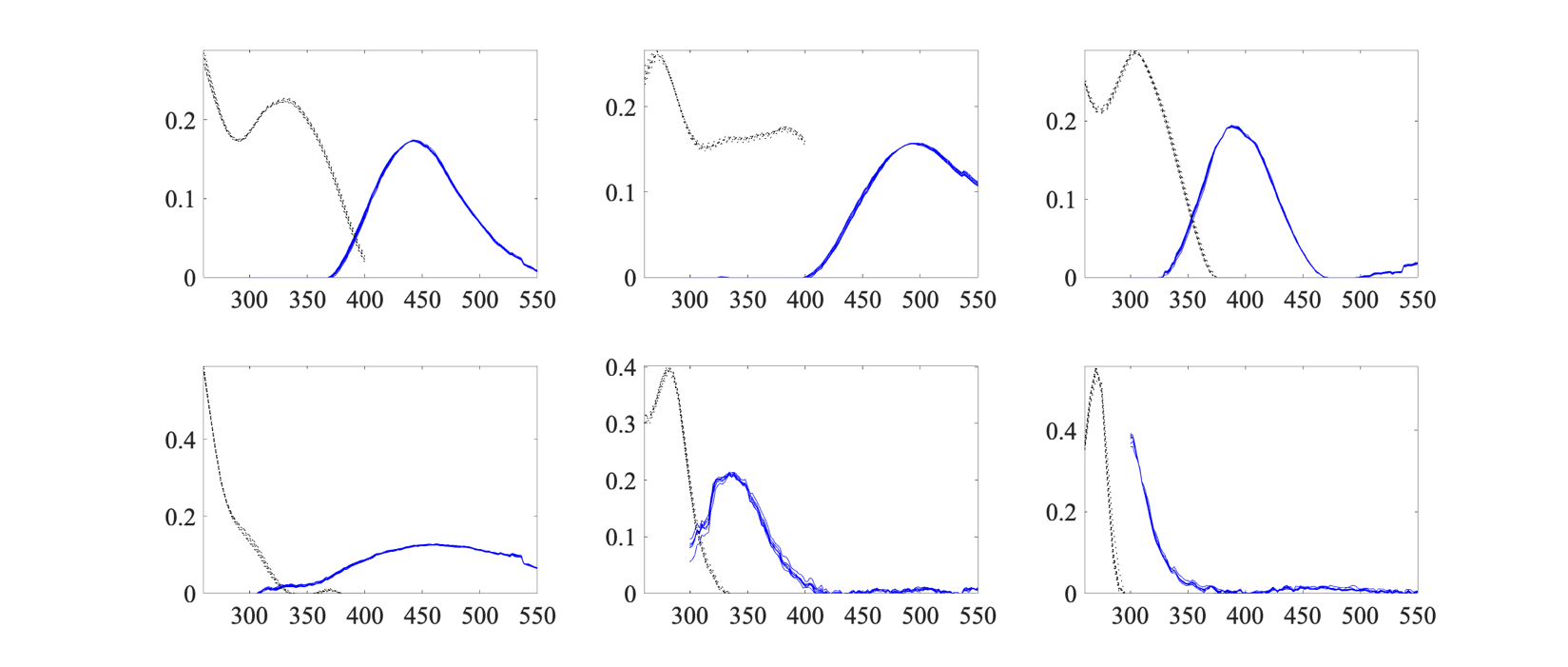
**

Figure S7. Split half analysis and validation of a six component PARAFAC model that explained >99.9% of total variation. *Top panel*: Six split halves showing consistency and validation of individual model components. *Bottom panel*: Overlap of all excitation and emission spectra shows consistency and validation of each component for all randomly split halves of the dataset.

Figure S8. Comparison of measured (red line) and modeled (blue line) irradiance for St. 10, following the description in the Supplemental Methods section.

**References**

1. Logozzo L, Tzortziou M, Neale P, Clark B. Photochemical and Microbial Degradation of Chromophoric Dissolved Organic Matter Exported from Tidal Marshes. *Journal of Geophysical Research: Biogeosciences* 2021.

2. Pickett JE, Neale PJ, Pickett JP. Annual solar spectral energy distributions in North America. *Polymer Degradation and Stability* 2020, **182:** 109380.
